# Comparison of [^18^F]AlF-NOTA-FAPI-04 PET/CT and [^18^F]FDG PET/CT for diagnosing lymph node metastasis in malignant tumors

**DOI:** 10.3389/fonc.2025.1605568

**Published:** 2025-07-24

**Authors:** Wanjing Zhou, Jiashun Dai, Kai Zheng, Xiang Peng, Yanyin Zhang, Chengzhi Jiang, Jian Yang, Hui Ye

**Affiliations:** Department of PET-CT Center, The Affiliated Cancer Hospital of Xiangya School of Medicine/Hunan Cancer Hospital, Central South University, Changsha, China

**Keywords:** [^18^F]AlF-NOTA-FAPI-04, [^18^F]FDG, PET/CT, fibroblast activation protein (FAP), lymph node metastases

## Abstract

**Purpose:**

This study compared the diagnostic performance, semi-quantitative capability, and staging accuracy of [^18^F]AlF-NOTA-FAPI-04 and [^18^F]FDG PET/CT in detecting lymph node metastases.

**Methods:**

This single-center retrospective study included 41 patients with suspected lymph node metastases who received both [^18^F]FDG and [^18^F]AlF-NOTA-FAPI-04 PET/CT. The study compared metastatic lymph node detection, semi-quantitative values, and N staging accuracy. Subgroup analyses were performed for lymph nodes with short-axis diameters (SADs) ≥10 and <10 mm.

**Results:**

A total of 41 patients with 126 nodes were included. [^18^F]AlF-NOTA-FAPI-04 outperformed [^18^F]FDG in the detection of metastatic lymph nodes, achieving higher accuracy in both patient-based (92.7% vs. 70.7%, *p* = 0.004) and node-based analyses (91.3% vs. 60.3%, *p* < 0.001). In semi-quantitative analysis, the maximum standardized uptake value and target-to-background ratio of [^18^F]AlF-NOTA-FAPI-04 were higher than those of [^18^F]FDG in metastatic lymph nodes (all *p* < 0.001). Both tracers distinguished metastatic from benign nodes with SAD ≥ 10 mm (*p* < 0.05). [^18^F]AlF-NOTA-FAPI-04 PET/CT could distinguish between benign and metastatic lymph nodes with SAD < 10 mm (*p* < 0.001), whereas [^18^F]FDG PET/CT could not (*p* > 0.05). [^18^F]AlF-NOTA-FAPI-04 also provided more accurate N staging assessments (87.8% vs. 65.9%, *p* = 0.006). [^18^F]AlF-NOTA-FAPI-04 PET/CT enabled the correct diagnosis of more lymph nodes, leading to a change in the therapeutic regimen for eight patients (19.5%).

**Conclusion:**

[^18^F]AlF-NOTA-FAPI-04 PET/CT demonstrated superior diagnostic performance, semi-quantitative capability, and N staging accuracy compared to [^18^F]FDG PET/CT, particularly for small metastatic lymph nodes (SAD < 10 mm), offering enhanced guidance for tumor staging.

## Introduction

Cancer is a leading cause of premature death and a significant barrier to improving life expectancy (1). Evaluating lymph node status is critical for both therapy selection and prognosis prediction (2–4). Ultrasound (US), computed tomography (CT), and magnetic resonance imaging (MRI) are widely used to evaluate lymph node status in malignant tumors (5). However, these conventional imaging modalities exhibit low sensitivity and fail to provide functional information regarding lymph nodes. [^18^F]FDG PET/CT, which provides both structural and metabolic insights into tumors, enables early lesion detection. [^18^F]FDG PET/CT has become a powerful imaging modality for detecting malignant tumors and metastases, and it is more sensitive than conventional imaging modalities (US, CT, and MRI) in detecting lymph node metastasis (6, 7). However, inflammation or lymphoid follicular hyperplasia can result in false-positive imaging, potentially leading to incorrect cancer staging and inappropriate medical intervention (8). Additionally, mucinous adenocarcinomas and signet ring cell carcinomas often demonstrate low [^18^F]FDG uptake, resulting in the frequent underestimation of lymph node staging (9, 10).

Fibroblast activation protein (FAP) is a type II transmembrane serine protease predominantly expressed on the surface of cancer-associated fibroblasts (CAFs) in the tumor stroma. Fibroblast activation protein inhibitors (FAPIs) are imaging agents that target FAP for PET imaging (11, 12). Since FAPI PET does not depend on cellular glucose uptake, background signals in the brain, liver, and gastrointestinal tract are minimal. The tumor stroma forms around malignant cells larger than 1–2 mm, providing the basis for targeted imaging using FAP (13). These properties result in high-contrast and high-sensitivity images. Previous studies have shown that [^68^Ga]FAPI is superior to [^18^F]FDG in detecting various tumors and metastatic lesions (11, 14–16). However, the limited production of ^68^Ga results in insufficient supply, affecting the availability of [^68^Ga]FAPI PET imaging for patients. Additionally, the short half-life of ^68^Ga (t_1/2_ = 68 minutes) necessitates more stringent scanning protocols and acquisition settings. These factors limit the clinical application of [^68^Ga]FAPI. ^18^F produced by cyclotron has a longer half-life (t_1/2_ = 109.8 minutes), and [^18^F]FAPI can overcome these limitations (17). The recently developed aluminum-[^18^F]-labeled 1,4,7-triazacyclononane-*N*,*N*′,*N*″-triacetic acid-conjugated FAP inhibitor 04 ([^18^F]AlF-NOTA-FAPI-04) effectively visualizes various primary tumors and quantitatively assesses tumor involvement in cancer patients. However, there are only few comparative studies on the use of [^18^F]AlF-NOTA-FAPI-04 PET/CT versus [^18^F]FDG PET/CT in diagnosing lymph node metastasis across various cancer types. Consequently, we hypothesize that [^18^F]AlF-NOTA-FAPI-04 could serve as an alternative to [^18^F]FDG for imaging lymph node metastasis in various malignancies. The aim of this study was to determine whether [^18^F]AlF-NOTA-FAPI-04 outperforms [^18^F]FDG in detecting metastatic lymph nodes.

## Materials and methods

### Patient selection

The study involving human participants was reviewed and approved by the Ethics Committee of Hunan Cancer Hospital (2021 New Medical Technology Expedited Review [No. 02]). Our study was registered with ClinicalTrials.gov (registration number: NCT06557590). The patients provided written informed consent to participate in this study. Written informed consent was obtained from the individual(s) for the publication of any potentially identifiable images or data included in this article. All procedures were conducted in accordance with the Declaration of Helsinki. All patients who underwent [^18^F]FDG and [^18^F]AlF-NOTA-FAPI-04 PET/CT for suspected lymph node metastasis were retrospectively reviewed in our hospital from September 2021 to October 2023. The inclusion criteria for patients were as follows: 1) histologically confirmed malignant tumor via biopsy or surgery, 2) consent to undergo both [^18^F]FDG and [^18^F]AlF-NOTA-FAPI-04 PET/CT, and 3) interval between [^18^F]FDG and [^18^F]AlF-NOTA-FAPI-04 PET/CT shorter than 2 weeks. The exclusion criteria were as follows: 1) therapy during the interval between [^18^F]FDG and [^18^F]AlF-NOTA-FAPI-04 PET/CT and 2) absence of pathological or imaging follow-up to confirm the diagnosis.

### PET/CT image acquisition

Patients fasted for 6 hours prior to the [^18^F]FDG PET/CT scan and had blood glucose levels below 11.1 mmol/L at the time of the [^18^F]FDG injection. They were instructed to consume 500 mL of water before PET/CT scanning to enhance the excretion of [^18^F]FDG and [^18^F]AlF-NOTA-FAPI-04 through the renal calyces and subsequent voiding. After receiving an injection of 3.70 MBq/kg of [^18^F]FDG or [^18^F]AlF-NOTA-FAPI-04, patients rested quietly for approximately 60 minutes before undergoing a PET/CT scan. The PET/CT images covered the area from the skull vertex to the upper thighs. PET/CT scans were conducted using dedicated PET/CT scanners (GE Discovery MI PET/CT, GE Healthcare, Milwaukee, WI, USA). The CT scan parameters included a tube voltage of 110 kV, 30–180 mAs with automated dose modulation, and a slice thickness of 3.75 mm. Following the CT scan, a PET scan was immediately performed in 3D acquisition mode with 5–6 bed positions, each with a duration of 2.0 minutes. All acquired data were transferred to the Advantage Workstation (AW 4.7, GE Healthcare, Milwaukee, WI, USA) for reconstruction using the Q.Clear algorithm. All patients were monitored for adverse events for up to 30 minutes post-examination.

### Image analysis

[^18^F]AlF-NOTA-FAPI-04 PET/CT and [^18^F]FDG PET/CT were independently reviewed by two nuclear medicine physicians with over 10 years of experience. In [^18^F]AlF-NOTA-FAPI-04 PET/CT, metastatic lymph nodes were defined as having tracer uptake values exceeding twice those of the ascending aorta blood pool. In [^18^F]FDG PET/CT, metastatic lymph nodes were defined as having tracer uptake values exceeding those of the liver. For semi-quantitative analysis, the volume of interest (VOI) was plotted on the lymph node, and the corresponding short-axis diameter (SAD) was measured. The maximum standard uptake value (SUVmax) was determined automatically, and the target-to-background ratio (TBR) was calculated as the SUVmax of the lesion divided by the mean standardized uptake value (SUVmean) of the ascending aorta blood pool. Blood pool or liver activity was measured in a spherical VOI with a diameter of 1 cm placed in the ascending aorta or liver.

### Reference standards

Histopathological results and follow-up imaging were used as standards for making the final diagnosis. For lymph nodes with pathological results, the original histopathological reports of formalin-fixed surgical specimens were reviewed. Lymph nodes were classified as metastatic if metastatic cancer cells were identified in ultrasound-guided core needle biopsies or surgically resected lymph node tissue biopsies. Nodes without detected metastatic cancer cells in tissue biopsies were classified as benign. For lymph nodes without pathological results, follow-up imaging should be conducted for at least 3 months (3–12 months) after PET/CT scans to determine the nature of lymph nodes. A reduction in diameter following treatment was regarded as a sign of malignant lymph nodes (18). Additionally, lymph nodes with increasing size and those with persistent and progressive changes listed below (irregular margin, inhomogeneous cortex, perifocal edema, absent fatty hilum, asymmetry in comparison to the contralateral site, contrast enhancement, and blurred nodal border) were considered malignant lymph nodes (6).

### Statistical analysis

The Kolmogorov–Smirnov test was used to assess the assumption of normal distribution. Continuous variables were expressed as mean and standard deviation when normally distributed or as medians and first (Q1) and third (Q3) quartiles when not normally distributed. Categorical variables were presented as counts and percentages. The diagnostic performance of [^18^F]FDG and [^18^F]AlF-NOTA-FAPI-04 PET/CT scans for detecting lymph node metastasis was analyzed using both patient-based and node-based methods to evaluate sensitivity, specificity, and accuracy. In the node-based analysis, the quantitative parameters of the nodes were compared between benign and metastatic nodes using the Mann–Whitney test. Sensitivity, specificity, and accuracy were compared using the McNemar test. Two-tailed *p* < 0.05 was considered to indicate a statistically significant difference. Statistical analyses were conducted using GraphPad Prism (V9.0, GraphPad Software Corporation) and MedCalc (V20.218, MedCalc Software Ltd.).

## Result

### Participant cohort

**Figure 1:**
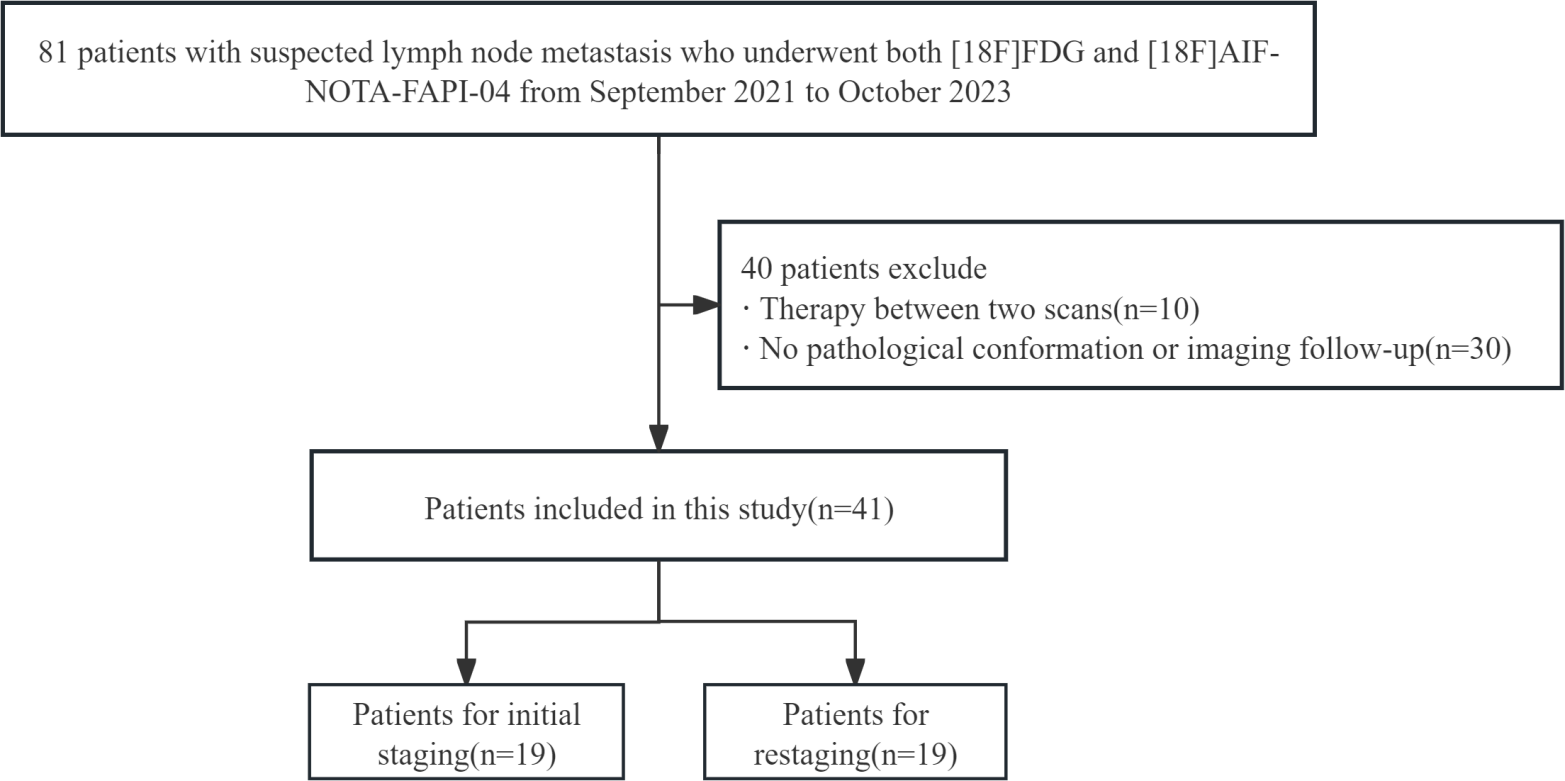
Flowchart of study design.

A total of 186 patients underwent [^18^F]AlF-NOTA-FAPI-04 PET/CT and [^18^F]FDG PET/CT to evaluate the stage of malignant tumors. Initially, 81 patients met the inclusion criteria. After excluding 40 patients, the remaining 41 patients were included in the final study. The cohort consisted of 13 men and 28 women, with a mean age of 52.4 ± 12.0 years. Of the 41 patients, 19 who were newly diagnosed with malignant tumors underwent PET/CT scans for initial staging. The remaining 22 participants underwent PET/CT for restaging. Patient characteristics are summarized in **Table 1**. The study flow diagram is presented in **Figure 1**.

**Table 1:** Characteristics of patients.

Characteristic	Value
Gender (male:female)	13 (31.7%): 28 (68.3%)
Age (years)	52.4 ± 12.0
Interval time (FDG PET–FAPI PET, days)	3.4 ± 3.6
Tumor entity	
Gastric cancer	15 (36.6%)
Thyroid cancer	6 (14.6%)
Breast cancer	5 (12.2%)
Liver cancer	3 (7.3%)
Lung cancer	3 (7.3%)
Colorectal cancer	3 (7.3%)
Nasopharyngeal carcinoma	1 (2.4%)
Throat cancer	1 (2.4%)
Ewing’s sarcoma	1 (2.4%)
Cervical cancer	1 (2.4%)
Ovarian cancer	1 (2.4%)
Pancreatic cancer	1 (2.4%)
Lymph node diagnosis	
Imaging follow-up	25 (60.9%)
Lymphadenectomy biopsy	14 (34.1%)
puncture biopsy	1 (2.5%)
Endoscopic surgery	1 (2.5%)
Indication	
Staging	19 (46.3%)
Restaging	22 (53.7%)
Short diameter	
≥10 mm	35 (27.8%)
<10 mm	91 (72.2%)

### Adverse events

All patients tolerated the [^18^F]AlF-NOTA-FAPI-04 PET/CT examination well. There were no signs of any [^18^F]AlF-NOTA-FAPI-04-related pharmacological or physiological effects. None of the patients reported any abnormal symptoms.

### Comparison of [^18^F]AlF-NOTA-FAPI-04 and [^18^F]FDG in diagnosis of metastatic lymph nodes

A total of 126 lymph nodes were identified in 41 patients, with 89 lymph nodes confirmed by follow-up imaging and 37 lymph nodes confirmed by pathological results. Among these, metastasis was confirmed in 87 lymph nodes in 27 patients. Lymph node involvement included 81 true-positive, six false-positive, five false-negative, and 34 true-negative findings with [^18^F]AlF-NOTA-FAPI-04 PET/CT, alongside 47 true-positive, 40 false-positive, 10 false-negative, and 29 true-negative findings with [^18^F]FDG PET/CT.

In the patient-based analysis, the sensitivity (96.2% vs. 74.1%, *p* = 0.031) and accuracy (92.7% vs. 70.7%, *p* = 0.004) of [^18^F]AlF-NOTA-FAPI-04 PET/CT in detecting metastatic lymph nodes were significantly higher than those of [^18^F]FDG PET/CT. However, there was no significant difference in specificity between [^18^F]FDG PET/CT and [^18^F]AlF-NOTA-FAPI-04 PET/CT in detecting metastatic lymph nodes (64.2% vs. 85.7%, *p* = 0.25). In the node-based analysis, the sensitivity (93.1% vs. 54.1%, *p* < 0.001) and accuracy (91.3% vs. 60.3%, *p* < 0.001) of [^18^F]AlF-NOTA-FAPI-04 PET/CT in detecting metastatic lymph nodes were also significantly higher than those of [^18^F]FDG PET/CT. However, the specificity (87.2% vs. 74.4%, *p* = 0.18) of [^18^F]AlF-NOTA-FAPI-04 PET/CT was not significantly higher than that of [^18^F]FDG PET/CT. The detailed comparison results of [^18^F]FDG and [^18^F]AlF-NOTA-FAPI-04 for detecting metastatic lymph nodes are shown in **Table 2**.

**Table 2:** Per-patient and per-node diagnosis of lymph node metastasis.

Analysis	Tracer	Sensitivity (%)	Specificity (%)	Accuracy (%)
Patient-based analysis	[^18^F]FDG	74.1% (20/27)	64.2% (9/14)	70.7% (29/41)
	[^18^F]AlF-NOTA-FAPI-04	96.2% (26/27)	85.7% (12/14)	92.7% (38/41)
	*p* value	0.031	0.25	0.004
Node-based analysis	[^18^F]FDG	54.1% (47/87)	74.4% (29/39)	60.3% (76/126)
	[^18^F]AlF-NOTA-FAPI-04	93.1% (81/87)	87.2% (34/39)	91.3% (115/126)
	*p* value	<0.001	0.18	<0.001

In the lymph node subgroup analysis with pathological results, a total of 37 lymph nodes from 16 patients were included. **Table 3** shows the accuracy of [^18^F]FDG PET/CT and [^18^F]AlF-NOTA-FAPI-04 PET/CT findings as validated by histopathology. The overall accuracy of [^18^F]AlF-NOTA-FAPI-04 PET/CT in lymph node assessment was significantly higher than that of [^18^F]FDG PET/CT in both patient-based and node-based analyses (patient-based: 93.8% vs. 50.0%, *p* = 0.016; node-based: 86.5% vs. 67.6%, *p* = 0.039). However, the sensitivity and specificity of [^18^F]AlF-NOTA-FAPI-04 PET/CT did not show statistically significant superiority over [^18^F]FDG PET/CT (sensitivity, patient-based: 90.0% vs. 50.0%, *p* = 0.125; node-based: 73.3% vs. 40.0%, *p* = 0.63; specificity, patient-based: 100.0% vs. 50.0%, *p* = 0.25; node-based: 95.5% vs. 86.4%, *p* = 0.625).

**Table 3:** The accuracy of [^18^F]FDG PET/CT vs. [^18^F]AlF-NOTA-FAPI-04 for patients with pathological results.

Analysis	Tracer	Sensitivity (%)	Specificity (%)	Accuracy (%)
Patient-based analysis	[^18^F]FDG	50.0% (5/10)	50.0% (3/6)	50% (8/16)
	[^18^F]AlF-NOTA-FAPI-04	90.0% (9/10)	100.0% (6/6)	93.8% (5/16)
	*p* value	0.125	0.25	0.016
Node-based analysis	[^18^F]FDG	40.0% (6/15)	86.4% (19/22)	67.6% (25/37)
	[^18^F]AlF-NOTA-FAPI-04	73.3% (11/15)	95.5% (21/22)	86.5% (32/37)
	*p* value	0.63	0.625	0.039

### Comparison of [^18^F]AlF-NOTA-FAPI-04 and [^18^F]FDG uptake in metastatic lymph nodes

**Figure 2:**
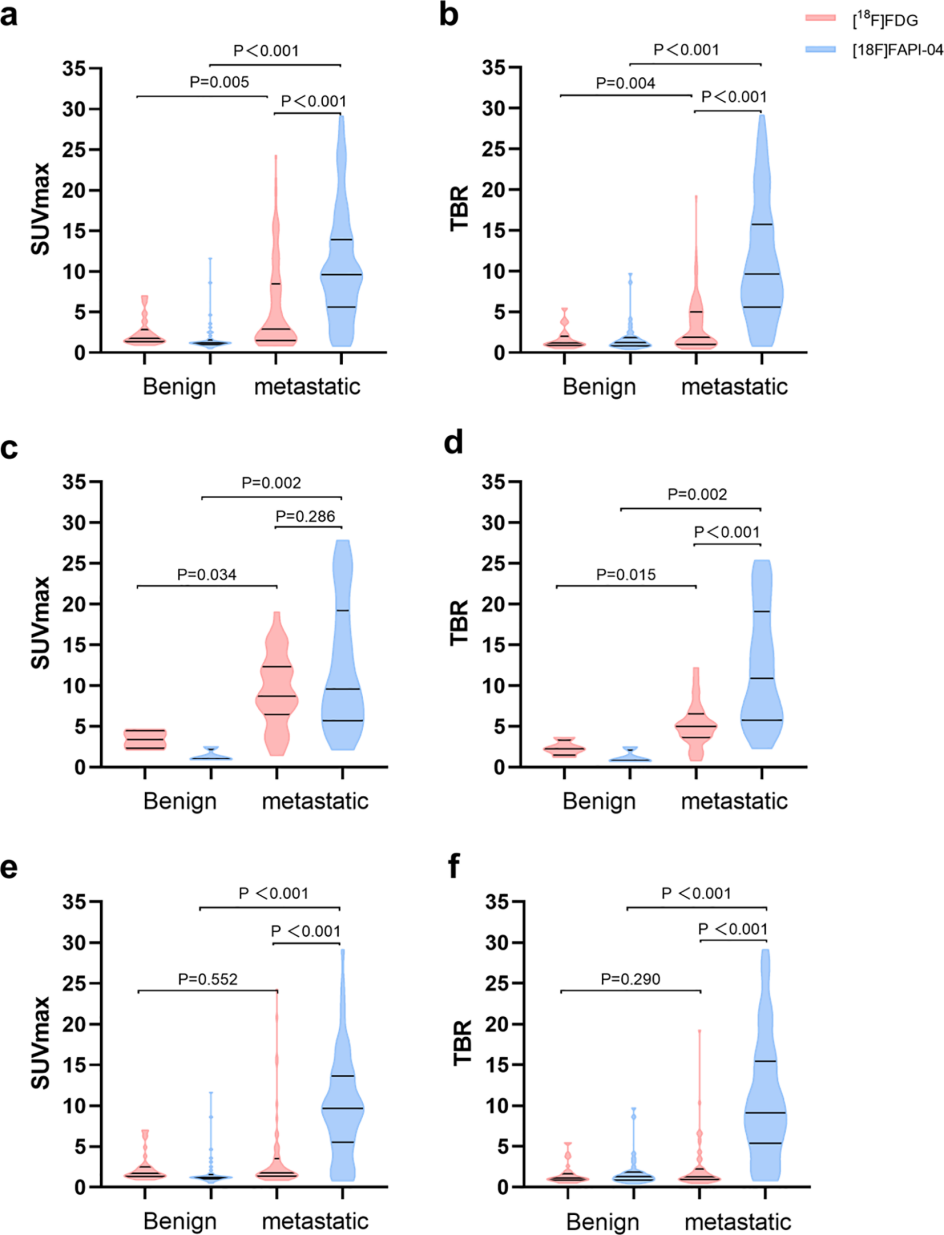
Comparison of SUVmax **(a)** and TBR **(b)** in 126 lymph nodes from 41 patients. Comparison of lymph node SUVmax **(c)** and TBR **(d)** in lymph nodes with SAD ≥ 10 mm. Comparison of lymph node SUVmax **(e)** and TBR **(f)** in lymph nodes with SAD < 10 mm. SUVmax, maximum standard uptake value; TBR, target-to-background ratio; SAD, short-axis diameter.

**Figure 3:**
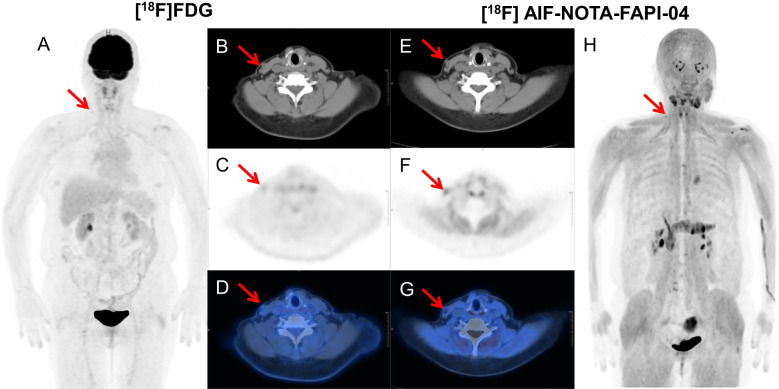
A 60-year-old woman with follicular thyroid cancer who had undergone total thyroidectomy 8 years ago. Her thyroglobulin level was elevated to 85.92 ng/mL. [^18^F]FDG PET/CT demonstrated a node with SAD of 6mm in right cervical level III with mild FDG uptake (SUVmax: 2.0, arrow). [^18^F]AlF-NOTA-FAPI-04 PET/CT showed moderate tracer accumulation in the corresponding region (SUVmax: 4.0, arrow). Subsequent lymph node biopsy revealed metastatic thyroid carcinoma. [^18^F]FDG PET/CT images **(A–D)** and [^18^F] AlF-NOTA-FAPI-04 PET/CT images **(E–H)**: maximum-intensity projections **(A, H)**, axial CT **(B, E)**, PET **(C, F)**, and fused PET/CT **(D, G)** are shown. SAD, short-axis diameter; SUVmax, maximum standard uptake value.

**Figure 4:**
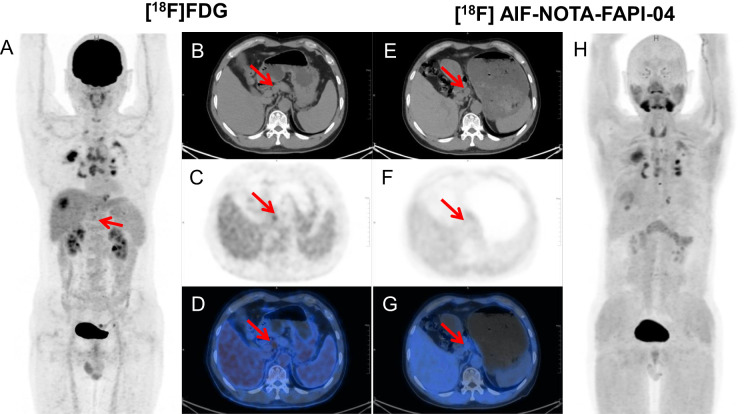
A 58-year-old male was incidentally found to have a right hepatic mass on CT scan with elevated alpha-fetoprotein (AFP, 234.47 ng/mL). [^18^F]FDG PET/CT demonstrated a lymph node with SAD of 15 mm around common hepatic artery with moderate [^18^F]FDG uptake (SUVmax 4.0, arrow). Subsequent [^18^F]AlF-NOTA-FAPI-04 PET/CT showed no significant tracer uptake in the corresponding region (SUVmax 1.2, arrow). The patient subsequently underwent right hepatic lesion resection with lymphadenectomy around common hepatic artery and hepatoduodenal ligament. Histopathological examination confirmed the station 8 lymph node as reactive hyperplasia. [^18^F]FDG PET/CT images **(A–D)** and [^18^F] AlF-NOTA-FAPI-04 PET/CT images **(E–H)**: maximum-intensity projections **(A, H)**, axial CT **(B, E)**, PET **(C, F)**, and fused PET/CT **((D, G)** are shown. SAD, short-axis diameter; SUVmax, maximum standard uptake value.

Metastatic lymph nodes showed significantly higher uptake of [^18^F]AlF-NOTA-FAPI-04 than [^18^F]FDG (SUVmax: 9.62 vs. 2.92, *p* < 0.001) (**Figure 2a**; **Table 4**), and the TBR from [^18^F]AlF-NOTA-FAPI-04 was also significantly higher than that of [^18^F]FDG (9.65 vs. 1.89, *p* < 0.001) (**Figure 2b**; **Table 4**). For lymph nodes with SAD ≥ 10 mm, there was no significant difference in [^18^F]AlF-NOTA-FAPI-04 and [^18^F]FDG uptake by metastatic lymph nodes (SUVmax: 9.57 vs. 8.71, *p* = 0.286) (**Figure 2c**; **Table 4**), but the TBR of [^18^F]AlF-NOTA-FAPI-04 was significantly higher than that of [^18^F]FDG (10.90 vs. 5.00, *p* < 0.001) (**Figure 2d**; **Table 4**). For lymph nodes with SAD < 10 mm, [^18^F]AlF-NOTA-FAPI-04 uptake in metastatic lymph nodes was significantly higher than [^18^F]FDG uptake (SUVmax: 9.67 vs. 1.75, *p* < 0.001) (**Figure 2e**; **Table 4**), and the TBR of [^18^F]AlF-NOTA-FAPI-04 was also significantly higher than that of [^18^F]FDG (9.13 vs. 1.27, *p* < 0.001) (**Figure 2f**; **Table 4**). Thus, [^18^F]AlF-NOTA-FAPI-04 is superior to [^18^F]FDG in demonstrating smaller metastatic lymph nodes. Representative cases showing the superiority of [^18^F]AlF-NOTA-FAPI-04 PET/CT over [^18^F]FDG PET/CT are presented in **Figures 3**, **4**.

**Table 4:** Uptake of [^18^F]FDG vs. [^18^F]AlF-NOTA-FAPI-04 by metastatic and benign lymph nodes.

Tracer	Benign lymph nodes		Metastatic lymph nodes		*p*-Value	
	SUVmax	TBR	SUVmax	TBR	*p*1	*p*2
[^18^F]FDG	1.78 [1.36, 2.84]	1.19 [0.88, 1.98]	2.92 [1.50, 8.47]	1.89 [1.00, 5.01]	0.005*	0.004*
[^18^F]AlF-NOTA-FAPI-04	1.21 [1.00, 1.55]	1.23 [0.82, 1.85]	9.62 [5.61, 13.94]	9.65 [5.57, 15.73]	<0.001*	<0.001*
p value			<0.001*	<0.001*		
Node with SAD ≥ 10 mm						
[^18^F]FDG	3.41 [2.31, 4.49]	2.25 [1.48, 3.31]	8.71 [6.45, 12.33]	5.00 [3.62, 6.56]	0.034*	0.015*
[^18^F]AlF-NOTA-FAPI-04	1.05 [0.94, 2.17]	0.84 [0.74, 2.08]	9.57 [5.69, 19.21]	10.90 [5.75, 19.10]	0.002*	0.002*
p value			0.286	<0.001*		
Node with SAD < 10 mm						
[^18^F]FDG	1.71 [1.32, 2.49]	1.11 [0.86, 1.65]	1.75 [1.36, 3.52]	1.27 [0.95, 2.24]	0.552	0.29
[^18^F]AlF-NOTA-FAPI-04	1.22 [1.04, 1.56]	1.31 [0.86, 1.85]	9.67 [5.53, 13.64]	9.13 [5.39, 15.43]	<0.001*	<0.001*
p value			<0.001*	<0.001*		

*p < 0.05.

*p*-value was used to compare the SUVmax and TBR of [^18^F]FDG with those of [^18^F]AlF-NOTA-FAPI-04.

*p*1 value was used to compare the SUVmax of [^18^F]FDG or [^18^F]AlF-NOTA-FAPI-04 between benign and metastatic lymph nodes.

*p*2 value was used to compare the TBR of [^18^F]FDG or [^18^F]AlF-NOTA-FAPI-04 between benign and metastatic lymph nodes.

### [^18^F]AlF-NOTA-FAPI-04 and [^18^F]FDG uptake of benign and metastatic lymph nodes

In [^18^F]AlF-NOTA-FAPI-04 PET/CT, the SUVmax and TBR of metastatic lymph nodes were significantly higher than those of benign lymph nodes (SUVmax, 9.62 vs. 1.21, *p* < 0.001; TBR, 9.65 vs. 1.23, *p* < 0.001) (**Figures 2a, b**; **Table 4**). Similarly, the SUVmax and TBR of metastatic lymph nodes were significantly higher than those of benign lymph nodes in [^18^F]FDG PET/CT (SUVmax, 2.92 vs. 1.78, *p* = 0.005; TBR, 1.89 vs. 1.19, *p* = 0.004) (**Figures 2a,b**; **Table 4**).

In the diagnosis of metastatic lymph nodes with SAD ≥ 10 mm, the [^18^F]AlF-NOTA-FAPI-04 and [^18^F]FDG uptake in metastatic lymph nodes was higher than that in benign lymph nodes (FAPI: 9.57 vs. 1.05, SUVmax, *p* = 0.002; TBR, 10.90 vs. 0.84, *p* = 0.002; FDG: SUVmax, 8.71 vs. 3.41, *p* = 0.034; TBR, 5.00 vs. 2.25, *p* = 0.015) (**Figures 2c, d**, **Table 4**).

In the diagnosis of metastatic lymph nodes with SAD < 10 mm, the SUVmax and TBR of [^18^F]AlF-NOTA-FAPI-04 were higher in metastatic lymph nodes than in benign lymph nodes (SUVmax: 9.67 vs. 1.22, *p* < 0.001; TBR: 9.13 vs. 1.31, *p* < 0.001) (**Figures 2e, f**; **Table 4**). However, there were no statistically significant differences in the SUVmax and TBR of [^18^F]FDG PET/CT between metastatic and benign lymph nodes (SUVmax: 1.75 vs. 1.71, *p* = 0.552; TBR: 1.27 vs. 1.11, *p* = 0.29) (**Figures 2e, f**; **Table 4**).

### Influence of [^18^F]FDG vs. [^18^F]AlF-NOTA-FAPI-04 PET/CT on N staging of patients

According to the follow-up results, the accuracy of [^18^F]AlF-NOTA-FAPI-04 PET/CT in predicting the N staging was 87.8% (36/41). The N staging was underestimated in 7.3% (3/41) and overestimated in 4.9% (2/41) of the patients. The accuracy of [^18^F] FDG PET/CT prediction in N staging was 65.9% (27/41). N staging was underestimated in 21.9% (9/41) of the patients and overestimated in 12.2% (5/41) of the patients. In evaluating the N staging of tumor patients, [^18^F]AlF-NOTA-FAPI-04 PET/CT was superior to [^18^F]FDG PET/CT (87.8% vs. 65.9%, *p* = 0.006). In the subgroup analysis of SAD ≥ 10 mm, benign and malignant lymph nodes in two patients were misdiagnosed on [^18^F]FDG PET/CT, resulting in an incorrect N staging. In the subgroup analysis of SAD < 10 mm, benign and malignant lymph nodes were misdiagnosed in 12 patients on [^18^F]FDG PET/CT, resulting in an incorrect N staging. In five patients, benign and malignant lymph nodes were misdiagnosed on [^18^F]AlF-NOTA-FAPI-04 PET/CT, resulting in an incorrect N staging. In [^18^F]AlF-NOTA-FAPI-04 PET/CT, only benign and malignant lymph nodes with SAD < 10 mm were misdiagnosed, resulting in an incorrect N staging.

### Changes in patient management

[^18^F]AlF-NOTA-FAPI-04 PET/CT enabled the correct diagnosis of more lymph nodes, leading to a change in the therapeutic regimen for eight patients (19.5%): two patients were changed from thyroid stimulating hormone (TSH) suppression therapy to cervical lymph node dissection surgery, two patients were changed from surgery to chemotherapy, one patient was changed from chemoradiotherapy to induction chemotherapy + chemoradiotherapy, one patient was changed from surgery to targeted therapy + chemotherapy, one patient was changed from primary lesion surgery to primary lesion surgery + radiotherapy for metastatic lymph nodes, and one patient was changed from surgery on the primary lesion + radiotherapy for metastatic lymph nodes to surgery on the primary lesion.

## Discussion

Our research indicated that [^18^F]AlF-NOTA-FAPI-04 exhibited superior sensitivity and accuracy in detecting metastatic lymph nodes of malignant tumors compared to [^18^F]FDG PET/CT. The semi-quantitative analysis revealed that the uptake of [^18^F]AlF-NOTA-FAPI-04 by metastatic lymph nodes was significantly higher than that of [^18^F]FDG PET/CT. Furthermore, our study demonstrated that [^18^F]AlF-NOTA-FAPI-04 PET/CT could identify metastatic lymph nodes with SAD < 10 mm, while [^18^F]FDG PET/CT struggled to do so. Patient-based analysis revealed that [^18^F]AlF-NOTA-FAPI-04 PET/CT improved sensitivity upstaged 22.1% of the patients without compromising specificity.

**Figure 5:**
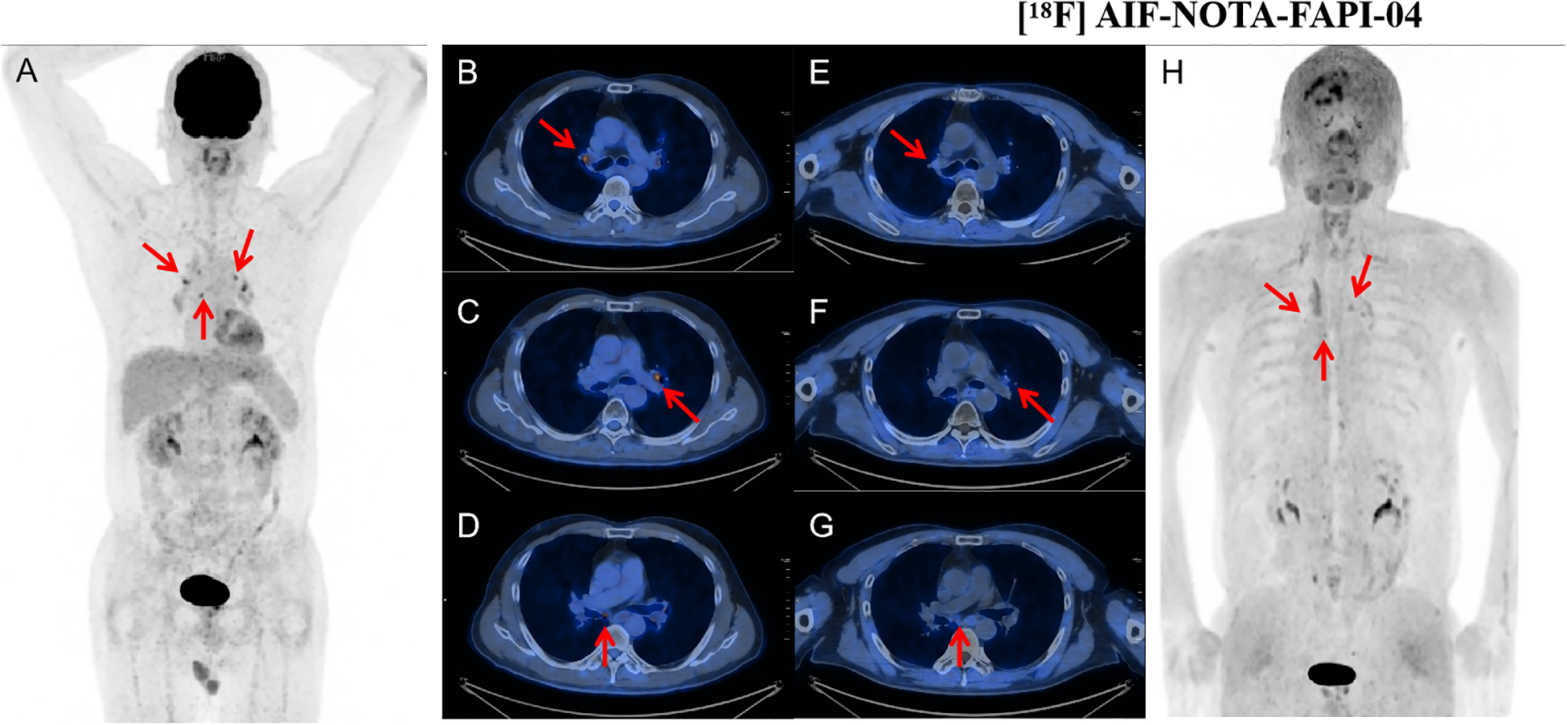
A 51-year-old man with lung adenocarcinoma who had undergone right superior lobe wedge resection 1 year ago. [^18^F]FDG PET/CT showed multiple lymph nodes in bilateral hilar (fused PET/CT: **B**, **C**, **E**, and **F**, arrow) and subcarinal zone (fused PET/CT: **D** and **G**, arrow) with high uptake and SUVmax of 6.3–7.0. [^18^F]AlF-NOTA-FAPI-04 PET/CT showed low-to-moderate uptake of lymph nodes at corresponding sites, SUVmax 2.5–3.6. Subsequent imaging showed no significant changes in the lymph nodes in the corresponding area, confirming that these lymph nodes were benign. [^18^F]FDG PET/CT images **(A–D)** and [^18^F]AlF-NOTA-FAPI-04 PET/CT images **(E–H)**: maximum-intensity projections **(A, H)**. SUVmax, maximum standard uptake value.

Previous studies have confirmed that [^18^F]FDG PET/CT has a major defect in detecting metastatic lymph nodes with low-to-medium sensitivity (such as head and neck cancer, cervical cancer, and gastrointestinal cancer) (19–21). Encouragingly, the high sensitivity of [^18^F]AlF-NOTA-FAPI-04 PET/CT for metastatic lymph nodes helps to address this limitation and expand the clinical application of PET/CT. Previous studies have shown that [^68^Ga]Ga-FAPI PET/CT is more sensitive than [^18^F]FDG PET/CT in detecting metastatic lymph nodes. Pang et al. conducted a post-hoc retrospective subgroup analysis of 35 gastrointestinal cancer patients and found that the sensitivity of [^68^Ga]FAPI PET/CT for detecting metastatic lymph nodes was significantly higher than that of [^18^F]FDG (79% vs. 54%, *p* < 0.001), although its specificity showed no statistically significant difference (82% vs. 89%, *p* = 0.50) (22). Wang et al. reported that [^68^Ga]FAPI PET/CT identified a greater number of metastatic lymph nodes in advanced lung cancer compared to [^18^F]FDG PET/CT (23). Our research shows that the sensitivity of [^18^F]AlF-NOTA-FAPI-04 PET/CT in detecting metastatic lymph nodes is higher than that of [^18^F]FDG, which is consistent with the results of previous studies. [^18^F]AlF-NOTA-FAPI-04 PET/CT is more sensitive in identifying metastatic lymph nodes than [^18^F]FDG PET/CT, attributable to its higher uptake and TBR. However, [^18^F]AlF-NOTA-FAPI-04 uptake was also observed in reactive lymph nodes in our study. Representative cases are shown in **Figure 5**. Previous studies have indicated the presence of certain false-positive lymph nodes in [^68^Ga]FAPI PET/CT results (24–26). This may be due to inflammation in the lymph nodes with reactive hyperplasia, and non-specific fibrosis induced by inflammation may also lead to the positive uptake of [^18^F]AlF-NOTA-FAPI-04 (27, 28). Therefore, when evaluating lymph node status with [^18^F]AlF-NOTA-FAPI-04 PET/CT, potential inflammatory uptake must be considered. Combining the clinical history and the drainage route of lymph nodes, as well as CT and magnetic resonance features, can help distinguish metastatic lymph nodes from inflammatory lymph nodes.

Accurately determining the status of lymph nodes is key to choosing the treatment plan and determining the degree of lymph node dissection. The Zhou et al. retrospective subgroup analysis of 35 non-small cell lung cancer patients further demonstrated markedly higher accuracy with [^68^Ga]FAPI-04 (0.94 vs. 0.30 for [^18^F]FDG) (29). Wu et al. conducted a meta-analysis comparing the detection rates of metastatic lymph nodes with [^68^Ga]Ga-FAPI PET/CT and [^18^F]-FDG PET/CT across various cancer types, and the results also showed that [^68^Ga]FAPI PET/CT outperforms [^18^F]FDG PET/CT in detecting lymph node metastasis ([^68^Ga]Ga-FAPI vs. [^18^F]FDG: 82% vs. 67%, *p* = 0.04) (30). Our study results were consistent with the findings of previous studies. The improvement in diagnostic accuracy may be attributed to the enhanced sensitivity in diagnosing metastatic lymph nodes without affecting specificity.

Previous studies have indicated that [^18^F]FDG PET/CT exhibits low detection rates for micrometastatic lymph node lesions (31, 32). Zhou et al. showed that patients with stage III colorectal cancer, exhibiting metastatic lymph nodes with a SAD of 7–10 mm, may experience therapeutic benefits from para-aortic lymph node dissection and chemotherapy (33). Therefore, the status of these non-enlarged lymph nodes may influence the choice of treatment or surgery. However, it is difficult to differentiate between benign and malignant lymph nodes with SAD < 10 mm in clinical practice (34, 35). Currently, conventional imaging modalities demonstrate limited diagnostic performance in detecting small lymph nodes with SAD < 10 mm. Notably, our study focused on the application of [^18^F]AlF-NOTA-FAPI-04 PET/CT in the detection of metastatic lymph nodes with SAD < 10 mm. In this study, [^18^F]AlF-NOTA-FAPI-04 PET/CT was able to visualize micrometastatic lymph nodes (SAD < 10 mm) that were missed by [^18^F]FDG PET/CT. Therefore, [^18^F]AlF-NOTA-FAPI-04 PET/CT may play a complementary role to [^18^F]FDG PET/CT in detecting small metastatic lymph nodes. We speculated that the low glucose metabolism of micrometastatic lymph nodes makes them difficult to detect via [^18^F]FDG PET/CT. However, the abundance of tumor stroma surrounding these micrometastatic lymph nodes, which contains tumor-associated fibroblasts, allows metastatic lymph nodes with a SAD < 10 mm to be visible on [^18^F]AlF-NOTA-FAPI-04 PET/CT. Therefore, [^18^F]AlF-NOTA-FAPI-04 PET/CT could detect metastatic lymph nodes that are not visible on [^18^F]FDG PET/CT.

In the evaluation of N staging, [^18^F]AlF-NOTA-FAPI-04 PET/CT demonstrated misclassification exclusively in distinguishing between benign and malignant lymph nodes with SAD < 10 mm, thereby influencing the N staging assessment of malignant tumors. In contrast, [^18^F]FDG PET/CT exhibited misdiagnoses for both lymph nodes with SAD ≥ 10 mm and SAD < 10 mm, leading to inaccuracies in N staging assessment. [^18^F]AlF-NOTA-FAPI-04 PET/CT outperforms [^18^F]FDG PET/CT in the detection of occult metastatic lymph nodes, leading to accurate N staging. This advancement enables clinicians to make more precise staging assessments and optimize treatment strategies. This minimized the risk of occult lymph node metastases and informed alternative treatment strategies for high-stage patients ineligible for surgery.

This study has several limitations that warrant consideration. First, the relatively limited patient population and the unequal distribution of tumor types within the study cohort were significant drawbacks that may have affected the generalizability of the findings. Hence, it is crucial to increase the number of participants for each type of malignancy in future studies to ensure a more representative sample. Second, the majority of patients was confirmed by follow-up evaluation rather than histology due to technical and ethical tissue restrictions. In light of these limitations, our research also utilized morphologic and functional imaging, as well as follow-up imaging results, as reference standards. These additional measures provided a comprehensive assessment. Third, more than one-third of the patients [15 of 41 (36.6%)] had gastric cancer with metastatic lymph nodes that typically exhibited low-to-moderate uptake of [^18^F]FDG but strong uptake of [^18^F]AlF-NOTA-FAPI-04. Consequently, the diagnostic performance of [^18^F]AlF-NOTA-FAPI-04 PET/CT may be exaggerated due to patient distribution bias. Prospective trials with larger patient populations are necessary to further evaluate the diagnostic performance of [^18^F]AlF-NOTA-FAPI-04 PET/CT.

## Conclusion

[^18^F]AlF-NOTA-FAPI-04 PET/CT showed superior detection efficiency, quantitative capability in assessing metastatic lymph nodes, and management of N staging in patients with cancers compared to [^18^F]FDG PET/CT, especially in the detection of metastatic lymph nodes with SAD < 10 mm. [^18^F]AlF-NOTA-FAPI-04 PET/CT can provide more valuable guidance for tumor staging than [^18^F]FDG PET/CT.

## Data Availability

The original contributions presented in the study are included in the article/supplementary material. Further inquiries can be directed to the corresponding authors.
